# Editorial: Vascular Adjustments in Cardiovascular Disorders

**DOI:** 10.3389/fphys.2021.777488

**Published:** 2021-10-25

**Authors:** Ana Paula Davel, Javier Blanco-Rivero, Luciana V. Rossoni

**Affiliations:** ^1^Department of Structural and Functional Biology, Institute of Biology, University of Campinas, Campinas, Brazil; ^2^Department of Physiology, School of Medicine, Universidad Autónoma de Madrid, Madrid, Spain; ^3^Department of Physiology and Biophysics, Institute of Biomedical Science, University of São Paulo, São Paulo, Brazil

**Keywords:** cardiovascular diseases, vascular remodeling, vascular dysfunction, atherosclerosis, hypertension, obesity

The vascular system is involved in the distribution of blood flow to organs and tissues, as well as in the blood pressure control. The role played by conductance and resistance vessels as well as by the specific vascular beds (e.g., cerebral, pulmonary, or mesenteric vasculature) is different. In addition, vessel reactivity and function adjust under physiological and pathological conditions. Local, humoral, and neural mechanisms contribute to regulate and integrate the heterogeneity of vascular function. Myogenic tone, endothelial cells, perivascular adipose tissue (PVAT) secretion and innervation, and components of the extracellular matrix are local mechanisms implicated in the regulation of vascular tone and structure, thereby controlling vascular resistance and compliance.

Cardiovascular diseases (CVD) are considered a major health problem worldwide and correspond to the main cause of mortality in developing and developed countries. There are multiple factors triggering and/or contributing to worsening of cardiovascular disorders. Among these, physical inactivity, gene signature, disturbances to the microbiome, and environmental factors such as unhealthy diet and contaminants can direct or indirectly induce vascular dysfunction, thereby contributing to the progression of CVD and, consequently, leading to end-organ target damage. It is well-known that a dysfunction of the mechanisms controlling vascular resistance and compliance is involved in the development of alterations on vascular tone and/or structural remodeling. These changes are pivotal to the pathophysiology of several cardiometabolic diseases, such as hypertension, heart failure, diabetes, liver cirrhosis and obesity.

In the present Frontiers Research Topic, several articles focused on mechanisms involved on the vascular adjustments in obesity and diabetes. Moraes et al. reviewed the participation of vascular transient receptor potential (TRP) channel's function. The TRP superfamily consists of a diverse group of non-selective cation channels, that are present in endothelial and vascular smooth muscle cells, PVAT and perivascular sensory nerves. These channels have been implicated in the regulation of vascular tone, vascular cell proliferation, vascular wall permeability, and angiogenesis. As reviewed, vascular TRP channel's function is important for the prevention of vascular complications and end-organ damage in the setting of obesity and diabetes, and dysfunction of these channels is associated with cardiometabolic diseases. Barp et al. discussed PVAT-derived factors [with special attention to nitric oxide (NO), reactive oxygen species (ROS), and renin-angiotensin system (RAS)] as a putative target for intervention in CVD. In line with this point of view, dos Reis Costa et al. elegantly suggested a compensatory enhancement of the anticontractile effect of PVAT in male mice fed a high-carbohydrate diet, which involves angiotensin receptors (Mas and AT_2_) activation, and both nNOS and iNOS signaling, leading to increased production of NO and H_2_O_2_, and the opening of potassium channels as well.

Focused on the intima layer of the vessel, the review article from Potje et al. highlighted recent findings about the activation of endothelial glycocalyx and caveolae enzymes that participate in the synthesis and release of NO and ROS, and the alterations that could impair vascular function in CVD such as hypertension and atherosclerosis. Regarding atherosclerosis, Silva et al. discussed the involvement of RAS components [angiotensin II, angiotensin converting enzyme type 2 (ACE-2), and angiotensin 1–7] in vascular function and inflammation, which are important determinants of atherogenesis. Adding one more piece in the puzzle of atherosclerosis, Vieira-Alves et al. reviewed the involvement of the α7-nicotinic acetylcholine receptor (α7nAChR) as a new and promising target for the treatment of vascular inflammation, a mechanism involved in the pathogenesis of atherosclerosis.

It is well-known that physical inactivity reduces population survival, both in the presence or in the absence of CVD, and exercise training is a relevant non-pharmacological approach that enhances life quality, cardiovascular function, and survival. In line with this, the cross-sectional study ACELA (Klonizakis et al.) reported that long-term aquatic exercise improved NO-mediated endothelial function at micro- and macro-circulatory levels, providing evidence for the protective role of aquatic exercise against CVD in older populations. Interestingly, Rentz et al., in an elegant way, demonstrated that aerobic exercise training slowed down the progression of atherosclerotic lesion and positively altered plaque feature and inflammation profile. These authors proposed that regular aerobic exercise induces epigenetic anti-inflammatory changes in bone marrow stem cells, attenuating atherosclerosis.

Hypertension is associated with high mortality, end-organ damage, and vascular complications. Inflammation, RAS, COX-products, ROS and reduced NO bioavailability are involved in vascular complications of hypertension and cardiometabolic diseases. In this context, Martínez-Casales et al. reviewed the role of the heme oxygenase-1 as a potential pharmacological approach in the hypertensive pathology, focusing on its expression in macrophages. Moreover, Delgado et al. investigated possible sex differences on vascular G protein-coupled estrogen receptors (GPER) signaling. These authors observed that GPER-induced relaxation response is similar in resistance arteries of male and female hypertensive rats; however, with differential participation of endothelial mediators. The arterial remodeling and stiffness, frequently associated with hypertension, has been recognized as an independent predictor of mortality. Miotto et al. elegantly performed proteomic analysis in aorta of hypertensive animals, focusing on proteins involved in arterial stiffness. These authors identified proteins differentially expressed in hypertensive and perindopril-treated animals, which may contribute to identify possible targets for the management of arterial stiffness.

Metals and endocrine disruptors are dangerous pollutants and risk factors for CVD by inducing oxidative stress. Simões et al. showed that exposure to lead, in lower doses than the reference values accepted, enhanced COX-2-derived contractile prostanoids, increasing vascular tone of resistance arteries and, consequently, blood pressure. In an interesting way, the nutritional supplementation with a bioactive egg white hydrolysate seemed to be useful to prevent the HgCl_2_-induced vascular dysfunction in resistance arteries (Escobar et al.). Furthermore, García-Arévalo et al. demonstrated in the heart that the exposure to bisphenol-A, an endocrine disruptor which potentiates protein malnutrition-induced hypertension and cardiovascular risk, induced an inward remodeling of intramyocardial arteries, as well as ventricular dysfunction.

There are multiple studies which analyze alterations of regional circulations in different pathologies. Regarding cerebral circulation, Caracuel et al. demonstrated an adaptive mechanism of cerebral vessels to hepatic encephalopathy that might collaborate to increase brain blood flow by higher NO and PGI_2_ release. Pulmonary hypertension is a health problem characterized by vasoconstriction and vascular remodeling, leading to higher pulmonary vascular resistance. Hu et al. reviewed the interplay between the heat shock protein 90 (Hsp90) dysregulation and different proteins involved in pulmonary hypertension development, shedding novel insights into the intrinsic pathogenesis and potentially novel therapeutic strategies for this important disease.

Given the above, the editors consider that the articles published in this Frontiers Research Topic of Vascular Physiology contribute to the understanding of pathophysiological mechanisms involved in several CVD, as well to identify potential targets to prevent or minimize the vascular complications associated with CVD (Figure 1).

**Figure 1:**
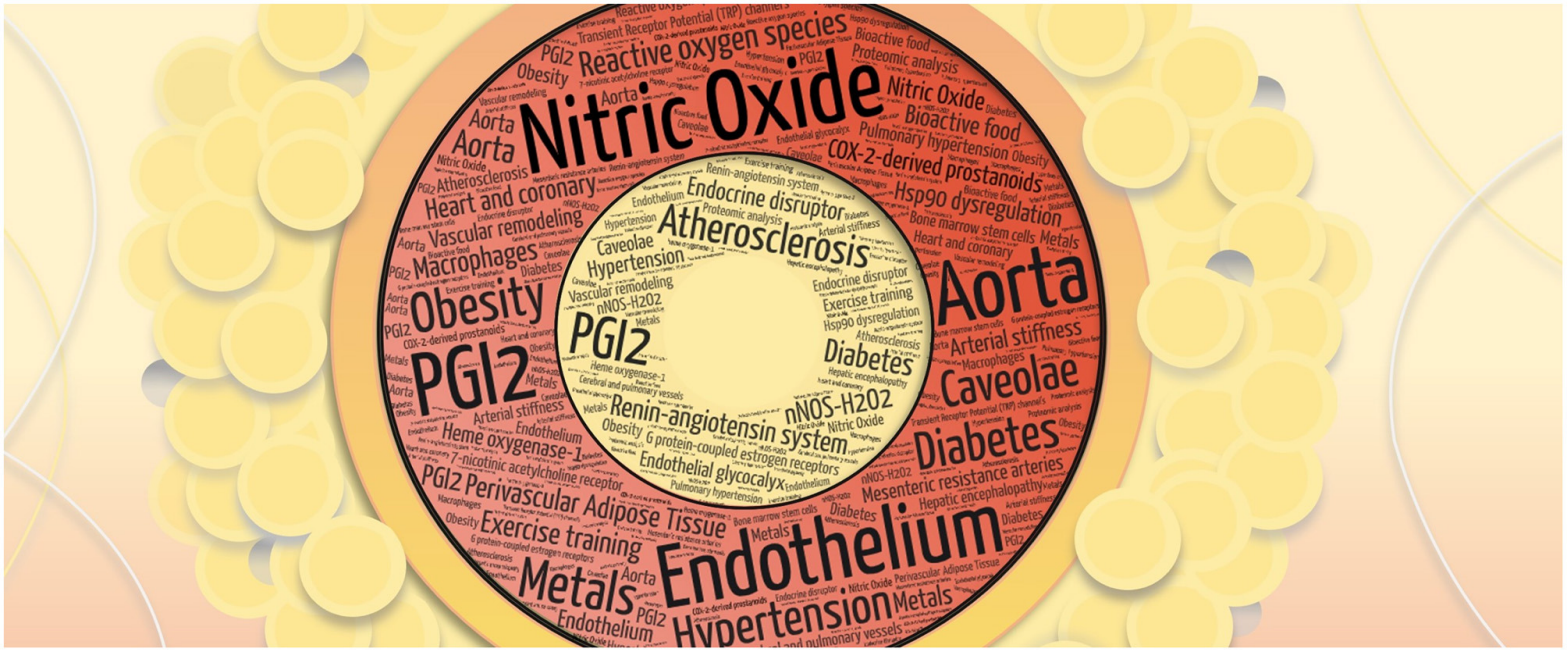
The keywords used in the articles published in this Frontiers Research Topic—*Vascular Adjustments in Cardiovascular Disorders*. Created using Word Cloud Art Creator.

## Author Contributions

LVR wrote the manuscript. APD and JB-R edited the manuscript. All authors contributed to the article and approved the submitted version.

## Funding

Supported by National Council for Scientific and Technological Development (CNPq, Brazil) grants 308682/2019-0 (to APD) and 306539/2017-9 (to LVR).

## Conflict of Interest

The authors declare that the research was conducted in the absence of any commercial or financial relationships that could be construed as a potential conflict of interest.

## Publisher's Note

All claims expressed in this article are solely those of the authors and do not necessarily represent those of their affiliated organizations, or those of the publisher, the editors and the reviewers. Any product that may be evaluated in this article, or claim that may be made by its manufacturer, is not guaranteed or endorsed by the publisher.

